# Analysis of tanshinone IIA induced cellular apoptosis in leukemia cells by genome-wide expression profiling

**DOI:** 10.1186/1472-6882-12-5

**Published:** 2012-01-16

**Authors:** Chang Liu, Jianqin Li, Liangjie Wang, Fuqun Wu, Linfang Huang, Yue Xu, Jieyu Ye, Bin Xiao, Fanyi Meng, Shilin Chen, Mo Yang

**Affiliations:** 1 Institute of Medicinal Plant Development, Chinese Academy of Medical Science, 151 MaLianWa North Road, Beijing, 100193, P.R.China; 2 Department of Hematology, Nanfang Hospital, Southern Medical University, Guangzhou, Guangdong 510515, P.R. China; 3 Institute of Hematology, Medical College, Jinan University, Guangzhou 510632, P.R.China; 4 LKS Faculty of Medicine, The University of Hong Kong, Hong Kong, P.R. China

**Keywords:** Gene expression profiling, apoptosis, CCL2, U-937 cell lines, tanshinone IIA (Tan IIA)

## Abstract

**Background:**

Tanshinone IIA (Tan IIA) is a diterpene quinone extracted from the root of *Salvia miltiorrhiza*, a Chinese traditional herb. Although previous studies have reported the anti-tumor effects of Tan IIA on various human cancer cells, the underlying mechanisms are not clear. The current study was undertaken to investigate the molecular mechanisms of Tan IIA's apoptotic effects on leukemia cells in vitro.

**Methods:**

The cytotoxicity of Tan IIA on different types of leukemia cell lines was evaluated by the 3-[4,5-dimethylthiazol-2,5]-diphenyl tetrazolium bromide (MTT) assay on cells treated without or with Tan IIA at different concentrations for different time periods. Cellular apoptosis progression with and without Tan IIA treatment was analyzed by Annexin V and Caspase 3 assays. Gene expression profiling was used to identify the genes regulated after Tan IIA treatment and those differentially expressed among the five cell lines. Confirmation of these expression regulations was carried out using real-time quantitative PCR and ELISA. The antagonizing effect of a PXR inhibitor L-SFN on Tan IIA treatment was tested using Colony Forming Unit Assay.

**Results:**

Our results revealed that Tan IIA had different cytotoxic activities on five types of leukemia cells, with the highest toxicity on U-937 cells. Tan IIA inhibited the growth of U-937 cells in a time- and dose-dependent manner. Annexin V and Caspase-3 assays showed that Tan IIA induced apoptosis in U-937 cells. Using gene expression profiling, 366 genes were found to be significantly regulated after Tan IIA treatment and differentially expressed among the five cell lines. Among these genes, CCL2 was highly expressed in untreated U-937 cells and down-regulated significantly after Tan IIA treatment in a dose-dependent manner. RT-qPCR analyses validated the expression regulation of 80% of genes. Addition of L- sulforaphane (L-SFN), an inhibitor of Pregnane × receptor (PXR) significantly attenuated Tan IIA's effects using colony forming assays.

**Conclusions:**

Tan IIA has significant growth inhibition effects on U-937 cells through the induction of apoptosis. And Tan IIA-induced apoptosis might result from the activation of PXR, which suppresses the activity of NF-κB and lead to the down-regulation of CCL2 expression.

## Background

Leukemia is one of the common malignant diseases. Artificial ionizing radiation, viruses, benzene, some petro-chemicals, and alkylating chemotherapy agents are now recognized as major causes of leukemia [1]. Approximately 80-100 million children and adults around the world develop some forms of leukemia each year. Identification of anti-leukemia therapies remains a top research priority. Recently, traditional Chinese herbal medicines have gained wide attention as alternative clinical options for the treatment of various malignant diseases, including leukemia, due to their antiviral, antioxidant, anti-inflammatory, and tumor apoptosis-inducing properties [2,3]. We are interested in the characterization of chemical compounds from these herbal medicines for further development.

Tanshinone IIA (Tan IIA) is a diterpene quinone extracted from the root of *Salvia miltiorrhiza *Bunge. The growth-inhibitory and apoptosis-inducing effects of Tan IIA on leukemia cells have recently been reported. For example, Tan IIA induced apoptosis in human leukemia cell lines HL-60 and K562 through the activation of caspase-3 [4]. Liu reported that the disruption of Δψm, activation of caspase-3, down-regulation of Bcl-2, survivin, and up-regulation of Bax were mainly responsible for Tan IIA-induced apoptosis on THP-1 cells [5]. In acute promyelocytic leukemia cells NB4, Tan IIA could promote cell differentiation and apoptosis with elevated C/EBP β and CHOP [6].

Tan IIA toxicities on other cancer lines have also been reported. Tan IIA could inhibit the growth of human hepatocellular carcinoma cells SMMC-7211 by apoptosis induction as a result of the up-regulation of P53, Fas and Bax, and the down-regulation of c-Myc and Bcl-2 [7]. Su suggested that the Tan IIA-induced apoptosis of breast cancer cells MDA-MB-231 may be attributed to the increased Bax to Bcl-xL expression ratios [8]. Lu reported that Tan IIA induced apoptosis in human breast cancer lines MCF-7 and MDA-MB-231 by decreasing the expression of P53 and Bcl-2 [9]. In HeLa cells, Tan IIA led cancer cells to G2/M phase arrest and subsequent apoptosis by disturbing the microtubule assembly [10,11]. In lung cancer A549 cells, Tan IIA-induced apoptosis was associated with a higher ratio of Bax/Bcl-2 [12].

The above studies have proposed different mechanisms of Tan IIA-induced apoptosis. The inconsistency in these proposed mechanisms may have resulted from the genetic diversities among the cell systems under study and the fact that the above studies focused on particular sets of genes or aspects. In the current paper, instead of focusing on a few candidate genes, we employed genome-wide expression profiling to identify the genes that are differentially expressed among leukemia cell lines exhibiting various Tan IIA sensitivities and significantly regulated after Tan IIA treatment as a way to elucidate the molecular mechanisms of Tan IIA in a systematic manner.

## Methods

### Reagents and cell culture conditions

The cell lines used in the current study include HL-60 (Human promyelocytic leukemia), U-937 (Human leukemic monocyte lymphoma), THP-1 (Human acute monocytic leukemia), MEG-01 (megakaryoblastic leukemia) and MOLT-4 (Human acute lymphoblastic leukemia). They were purchased from American Type Culture Collection (ATCC, Manassas, VA, USA) and cultured according to the manufacturer's instructions. Tan IIA (National Institute for the Control of Pharmaceutical and Biological Products, P.R.China) was dissolved in DMSO solution (0.01%). The stock concentration of Tan IIA was 200 μg/mL. L-sulforaphane (L-SFN) was purchased from Sigma-Aldrich (Shanghai, China) Trading Co., Ltd.

### Cytotoxicity assay for various cancer cell lines

Cell viability was evaluated by the 3-[4, 5-dimethylthiazol-2, 5]-diphenyl tetrazolium bromide (MTT) assay in triplicate [13]. For the experiments comparing the five cell lines, each cell line (2.5 × 10^5 ^cells/well) was cultured in the recommended medium in 96-well plates for 24 h. The culture medium was then removed, and the cells were treated with 0.1% DMSO as vehicle control or Tan IIA at 30 μg/mL. At the end of the cultivation, 20 μl of MTT working solution was added to the wells, which were incubated for an additional 4 h at 37°C. Finally, the absorbance of each well was measured using a micro-titer plate reader (TECAN, Vienna, Austria) at 570 nm. For the dose- and time-dependent experiments, the U-937 cells were treated with Tan IIA at concentrations of 1, 2, 3, 5 and 10 μg/mL for 0, 12, 24, 36 and 48 h, respectively.

### Detection of Tan IIA-induced apoptosis in U-937 cells using Annexin V and Caspase-3 assays

The assays were performed as described previously [14]. Briefly, the U-937 cells were maintained in DMEM medium plus 10% fetal bovine serum. Tan IIA (3 μg/mL) was added and the cells were cultured for 12, 24, 36 and 48 h, respectively. The vehicle control groups were treated with 0.1% DMSO. Apoptotic cell death was examined using annexin V-FITC/PI and active caspase-3-PE reagent kits (BD Biosciences, San Diego, CA, USA) according to the manufacturer's instructions. Ten thousand events were acquired for each sample and analyzed by flow cytometry using Lysis II software (FACScan; BD Pharmingen).

### GeneChip^® ^hybridization

To determine the expression levels of genome-wide gene transcripts in Tan IIA-treated and control cells, RNA was extracted using RNA extraction kit (Ambion, TX) according to manufacturer's instructions. Five μg of high-quality total RNA per sample was first converted to double-stranded cDNA. Biotin-labelled cRNA was subsequently synthesized on cDNA templates and fragmented prior to hybridization to Affymetrix GeneChip HG-U133 plus 2 slides following the manufacturer's recommendations (Affymetrix, Santa Clara, CA, USA). The integrity of cDNA, cRNA, and fragmented cRNA was assessed by running aliquots on the Bioanalyzer (Agilent Technologies Inc., Palo Alto, CA, USA). Hybridized probes were detected with streptavidin-phycoerythrin and scanned on an Affymetrix GeneChip Scanner 3000 (Affymetrix, Santa Clara, CA, USA).

### Microarray data analysis

Microarray images were visually inspected for hybridization artifacts and then analyzed with Affymetrix GeneChip Operating Software (GCOS) according to Affymetrix's guidelines (http://www.affymetrix.com). Affymetrix Microarray Suite MAS 5.0 algorithm was used for intensity estimation and data normalization. The cell intensity files (.cel) of all arrays were also analyzed using dChip software [15], which employs the invariant set normalization method to normalize the arrays. In addition, the PM-only model was used to detect the less-abundant genes in a more sensitive fashion [16,17]. Functional annotation of genes was performed at the NETAFFX Analysis Center. MAPPFinder [18] was used to find all pathway maps containing the significantly differentially expressed genes (SDEGs). The GeneChip data have been submitted to NCBI's Gene Expression Omnibus with accession number GSE33358.

### Real-time quantitative Polymerase Chain Reaction (RT-qPCR)

U-937 cells were treated with Tan IIA at 3 μg/mL and cultured for 1, 2, 4, 6, 12, 24 and 36 h. Total cellular RNA was extracted using Trizol reagent (Invitrogen Corp., Carlsbad, CA, USA). The cDNAs were synthesized using M-MLV reverse transcriptase with oligo-d (T) 15 primers according to the manufacturer's instructions (Promega, Madison, WI, USA). The primers used to detect the expression of target genes are listed in Additional file 1, Table S1. *ABL *was used as internal control. RT-qPCR was performed with SYBR-Green I intercalating dye (Bio-Rad, Hercules, CA, USA) using a MyiQ Single Color Real-time PCR Detection System (Bio-Rad). The following PCR program was used: an initial denaturation at 95°C for 5 min, 40 cycles of 95°C for 15 s, 60°C for 15 s and 72°C for 20 s. The RT-qPCR data were analyzed using the MyiQ software (Bio-Rad). All experiments were independently repeated three times.

### Enzyme-linked immunosorbent assay (ELISA)

U-937 cells were treated with Tan IIA at different concentrations of 0, 1, 3, 6 and 18 μg/mL for 12 h and harvested for ELISA. The expression level of CCL2 was determined using an ELISA kit (R & D, Minneapolis, MN, USA) following the manufacturer's instructions. A monoclonal antibody specific for CCL2 was pre-coated onto a microplate. Standards and samples were added to the wells. After washing away the unbound substances, enzyme-linked polyclonal antibodies specific for CCL2 were added to the wells. Subsequently, substrates were added. The colors developed were in proportion to the amount of CCL2 bound on the wells. The optical density of each well was measured using a microreader (DNATECH MR 5000) at a wavelength of 450 nm.

### U-937 Colony-Forming Unit (CFU) Assay

The assay was performed as described previously [14]. Colony-forming unit-U937 were cultured in methylcellulose (1%) supplemented with fetal calf serum (FCS, 30%), 1% BSA, 0.1 mM ß-mercaptoethanol, and with or without Tan IIA (2 ug/ml), PXR inhibitor L-sulforaphane (L-SFN) (1 uM) [19]. U937 cells (1 × 10^3 ^cells/mL) were seeded in triplicate and incubated for 3 days. The colony was considered as a cluster of 10 or more cells. Colonies were scored blindly.

### Statistical analysis

All experiments were performed in triplicate. The results were expressed as mean ± SD. Correlation analyses were performed using JMP software (version 6, SAS, NC, USA).

## Results

### Cytotoxicity of Tan IIA on various leukemia cell lines

**Figure 1:**
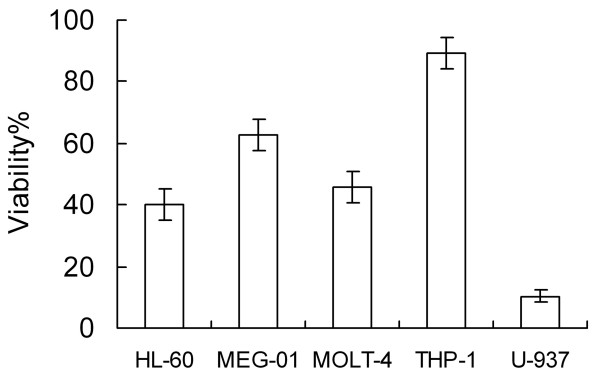
**Inhibitory effects of Tan IIA on five leukemia cell lines**. After the cells were treated with 30 μg/mL of Tan IIA for 24 h, MTT assay was used to detect cell viability rate.

Previous studies show that Tan IIA's effects are cell-specific. To identify a cell line sensitive to Tan IIA treatment as the model system for further study, five cell lines, namely, HL-60, MEG-01, MOLT-4, THP-1 and U-937 cell lines, were treated with Tan IIA at 30 μg/mL for 24 h. We observed that Tan IIA displayed different cytotoxic activities on the five types of leukemia cells (Figure 1). Compared with the control group (treated with 0.1% DMSO), the viability rates of HL-60, MEG-01, MOLT-4, THP-1 and U-937 cells were 40.0%, 62.5%, 45.6%, 89.5% and 10.3%, respectively. The corresponding cytotoxicity of Tan IIA on U-937 cells was significantly higher than that of the other cell lines; thus, further studies were conducted on these cells.

### Specific growth-inhibitory effects of Tan IIA on the U-937 cells

**Figure 2:**
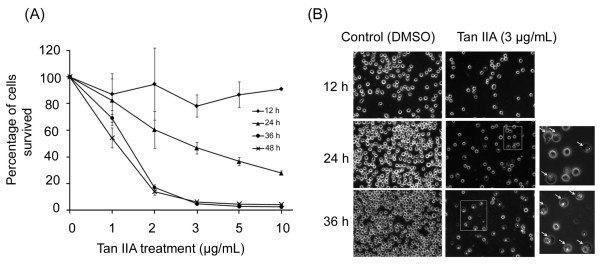
**Inhibitory effects of Tan IIA on the proliferation of U-937 cells**. (A) The percentage of cells that survived gradually decreased in a time- and dose-dependent manner after Tan IIA treatment. (B) Morphological changes of U-937 cells without (DMSO) and with Tan IIA treatment. The U-937 cells were treated with Tan IIA and the morphology of cell apoptosis was examined by phase-contrast microscopy. The magnification of the squared regions is shown to the right. Several cells undergoing apoptosis are indicated by arrows.

To determine if the effects of Tan IIA on U-937 were specific, U-937 cells were treated with Tan IIA at different concentrations of 1, 2, 3, 5 and 10 μg/mL for 12, 24, 36 and 48 h, respectively. As shown in Figure 2A, Tan IIA at concentrations over 2 μg/mL induced apoptosis when cultured with U-937 cells after 24-48 h. The percentage of cells that survived gradually decreased in a time- and dose-dependent manner. The morphologies of the apoptotic cells are shown in Figure 2B. Cellular surface alterations of U-937 cells were clearly observed after treatment with 3 μg/mL of Tan IIA for 24-36 h under phase-contrast microscopy. As indicated by arrows in Figure 2B, apoptotic cells exhibited rounding, shrinkage and blebbing of the plasma membrane, all of which are hallmarks of apoptosis.

### Test of Tan IIA-induced apoptosis in U-937 cells using Annexin V assay

**Figure 3:**
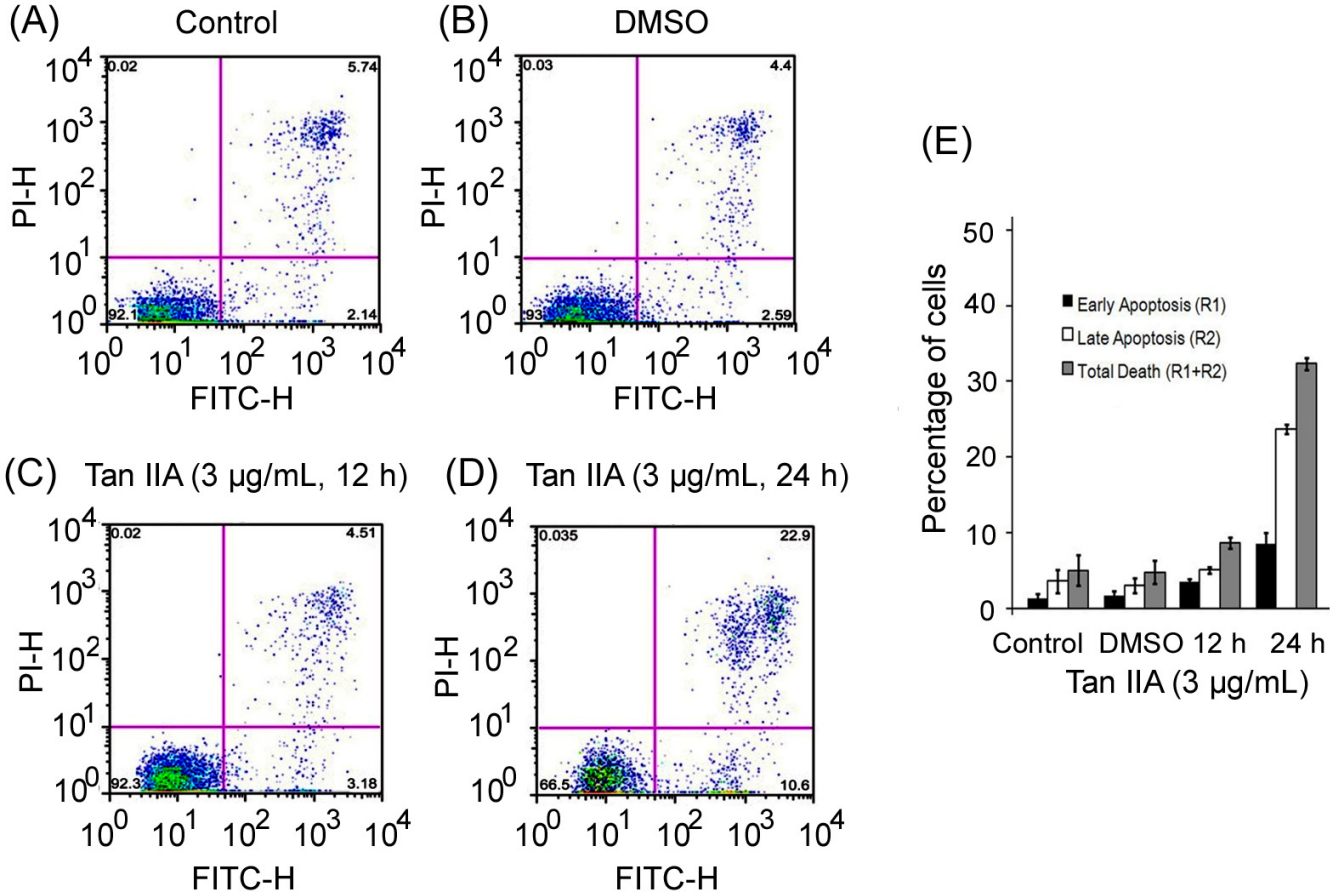
**Apoptotic effects of Tan IIA on U-937 cells analyzed by Annexin V assay**. Cells were treated with Tan IIA at 3 μg/mL and cultured for various time. The cells were stained with Annexin V-FITC and Propidium Iodide (PI) and subjected to flow cytometry analyses. Dot plots of control samples (A), DMSO-treated samples (B), samples treated with Tan IIA for 12 h (C), and samples treated with Tan IIA for 24 h (D) are shown. (E) Depicts the quantification and statistical analyses of the treatment effects. Percentages of early (R1, FITC+ and PI-), late (R2, FITC+ and PI+) and total (R1+R2, FITC+) apoptotic cells are shown. Experiments were repeated three times. Bars denote the standard error of the mean.

To further confirm the apoptotic effects of Tan IIA on U-937 cells, we measured the percentage of cells undergoing apoptosis after Tan IIA treatment using Annexin V assay. As shown in Figure 3, the amount of apoptotic cells gradually increased after treatments with Tan IIA at 3 μg/mL for 12-24 h. Figure 3E indicates that treatments with Tan IIA for 24 h significantly increased apoptotic cell populations in R1 (annexin V positive, PI negative, cells at the early phase of apoptosis), R2 (annexin V positive, PI positive, cells at the late phase of apoptosis), as well as R1+R2 (annexin V positive, total number of cells undergoing apoptosis).

### Test of Tan IIA-induced apoptosis in U-937 cells using Caspase 3 assay

**Figure 4:**
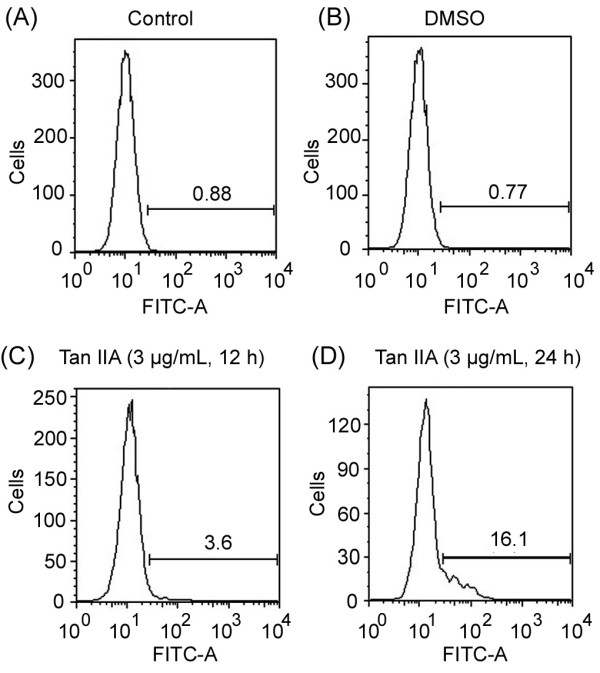
**Apoptotic effects of Tan IIA on U-937 cells analyzed by Caspase 3 assay**. U-937 cells are treated as described above, stained with Caspase 3-PE (FL-2) and subjected to flow cytometry analysis. Panels (A)-(D) show the histograms of control samples, DMSO-treated samples and Tan IIA treated samples for 12 h or 24 h respectively. Experiments were repeated three times. Bars denote the standard error of the mean.

We also measured the percentage of cells showing an elevated activity of caspase-3 in U-937 cells after Tan IIA treatment. Caspase 3 is a downstream effector protein of apoptosis and its elevated activity is indicative of cells undergoing apoptosis. As shown in Figure 4, the percentage of cells expressing caspase-3 in the control and DMSO-treated samples are similar. However, the samples that have been treated with Tan IIA for various times had significantly higher percentages of cells detected with caspase-3 activity. Our results confirmed that Tan IIA induces apoptosis in U-937 cells.

### Genome-wide expression profiling of U-937 cells treated with Tan IIA

To identify the potential molecular targets of Tan IIA, we employed Affymetrix GeneChip to identify genes that are up- and down-regulated in Tan IIA-treated U-937 cells. After preprocessing the raw data, the ratio of the intensity for each probe from samples treated with Tan IIA to that from untreated samples were calculated. The ratio was further log transformed using base 2 to generate the log_2_(fold change). All probe sets were then ranked based on the log_2 _(fold change). Genes that had log_2 _(fold change) greater than 1 or less than -1 after 12 h or 24 h of Tan IIA treatment were initially selected. These include 366 significantly differentially expressed genes (SDEGs) (Additional file 1, Table S2). From the annotation, many of these genes were found belonging to cell-cycle control, apoptosis pathways. Among the genes related to the apoptosis pathway, chemokine ligand 2 (CCL2, also named monocyte chemoattractant protein-1, MCP-1) is the most significantly down-regulated gene, whose expression has been down-regulated to -3.06- and -3.64-fold in the log scale after 12 and 24 h treatment, respectively. This correspondeded to ~10-fold down-regulation.

To include more genes for apoptosis-related pathways analysis we loosened the selection criteria and the genes that were differentially expressed with absolute fold change > 1.5 were further analyzed. Genes involved in apoptosis, NF-κB cascade and cell proliferation pathways are showed in Additional file 1, Table S3.

### Genome-wide expression profiling of five untreated cell lines

To identify genes that are related to U937's sensitivities to Tan IIA, we carried out expression profiling using U133 human GeneChips on five untreated cell lines described in Figure 1. After preprocessing the data, we performed the correlation analyses, in which the correlation between the Tan IIA's toxicity (represented by the percentage of cells that survived after treatment with Tan IIA at 30 μg/mL for 24 h) and the expression levels across the five different cell lines for each probe set was calculated. All probe sets were then sorted based on the correlation coefficient. The correlations for the probe sets significantly differentially expressed after Tan IIA treatment are shown in Additional file 1, Table S4. Compared with the previous experiments, CCL2 has a correlation coefficient of -0.788 and another gene, BCL2A1 has a correlation coefficient of -0.85. It should be pointed out that due to the diverse genetic background of the cell lines, different sets of genes might respond to Tan IIA treatment in these different cell lines. However, we think these results, when combined with those of other experiments, could still provide useful information for genes whose expression level responding to Tan IIA treatment.

### Validation of GeneChip data by RT-qPCR analysis

**Figure 5:**
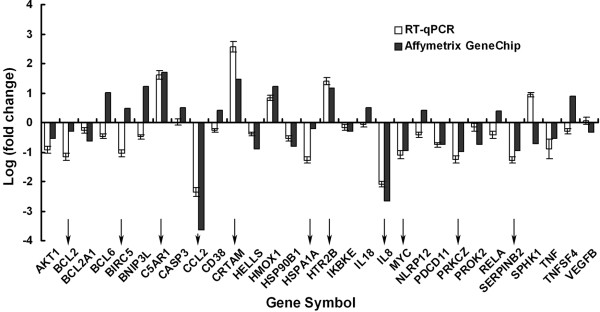
**Tan IIA induces mRNA expression of genes involved in apoptosis-related pathways**. Graphic representation of mRNA expression for the indicated genes at 24 h after Tan IIA treatment (3 μg/mL), as measured using RT-qPCR or Affymetrix GeneChip. Bars denote the standard error of the mean. Genes that have log_2 _(fold change) greater than 1 or less than -1 are indicated with arrows.

To validate the GeneChip results, we selected 30 genes that are involved in apoptosis-related pathways from Additional file 1, Table S3 for RT-qPCR analysis. The results are shown in Figure 5. As shown, three genes are up-regulated > 1 in the log_2 _(fold change) scale after Tan IIA treatment. And eight genes including *CCL2, BIRC5 *(Survivin), *BCL2, IL8, HSPA1A, MYC, PRKCZ *and *SERPINB2 *are down-regulated for more than one fold change (-1 in the log_2 _(fold change) scale). In total, we found that the directions of expression change for 80% of genes are consistent between GeneChip data and RT-qPCR data.

### RT-qPCR and ELISA analyses of CCL2 mRNA and protein expression in U-937 cells treated with Tan IIA

**Figure 6:**
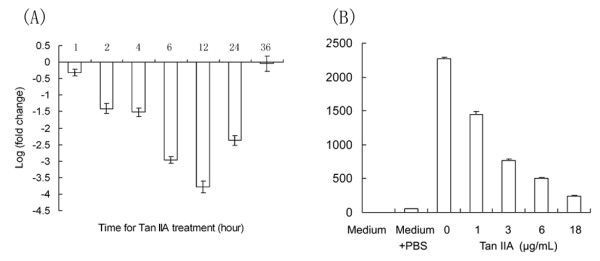
**Dose dependent regulation of CCL2 expression by Tan IIA**. (A) Regulation of CCL2 mRNA expression analyzed by RT-qPCR experiments. U-937 cells were treated as described in methods. Transcript levels were measured by RT-qPCR using *BAL *as an internal reference. Bars denote the standard error of the mean. All experiments were repeated three times. (B) Regulation of CCL2 protein expression determined by ELISA. U-937 cells were treated as described in methods. Experiments were repeated three times. Bars denote the standard error of the mean.

CCL2 that plays a role in the apoptosis pathway was one of the most significantly down-regulated genes; hence, we decided to study CCL2 further. To confirm the down-regulation of CCL2 expression in U-937, we examined the expression patterns of CCL2 in U-937 cells treated with Tan IIA using RT-qPCR and ELISA analyses. Our results showed that the mRNA levels of CCL2 in treated cells were lower than those of the control (Figure 6A). The CCL2 expression level clearly decreased after treatment with Tan IIA treatments for 2 h and reached the lowest level at 12 h, more than 10-fold down-regulation. The results of ELISA also confirmed the down-regulation of CCL2 expression in the treated cells at the protein level. We found that the CCL2 concentration in treated cells gradually decreased from 2273.33 pg/mL in the untreated cells to 1450 and 245 pg/mL after Tan IIA treatments at 1.0 and 18.0 μg/mL for 12 h, respectively (Figure 6B). These results indicate that Tan IIA significantly inhibit the expression of CCL2 at mRNA and protein levels.

### Antagonizing Tan IIA activity by PXR inhibitor L-SFN

**Figure 7:**
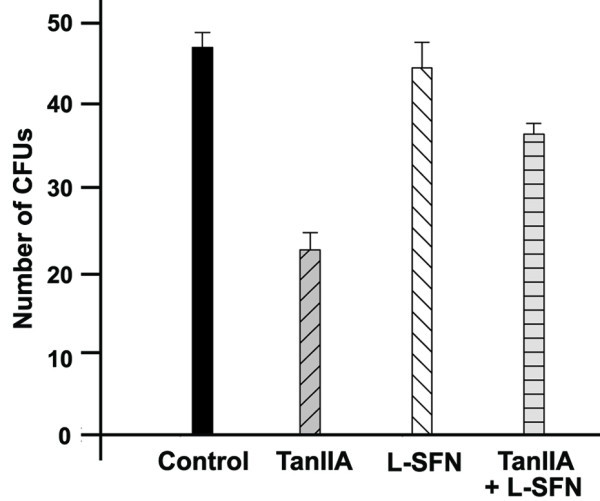
**PXR inhibitor L-SFN alleviates Tan IIA's inhibitory effect of the formation of Colony Forming Units (CFU)**.

Previous study suggests that one of Tan IIA's direct targets is PXR [20]. To test if Tan IIA indeed acts through PXR, we tested the colony forming efficiency in the presence and absence of Tan IIA and L-SFN (Figure 7). As shown, compared with that of the control (47 ± 1.83), Tan IIA reduced the number of colony forming units to 23 ± 2.1. L-SFN alone had little effects on the number of CFUs (44.5 ± 3.1). However, addition of L-SFN significantly mitigated the effect of Tan IIA, reducing colony forming units to 36.5 ± 1.3. The results suggested that L-SFN antagonizes Tan IIA's effect.

## Discussion

In the present study, we aimed to systematically investigate the molecular mechanisms underlying the growth inhibitory effect of Tan IIA on leukemia cells. We found that Tan IIA displays significant cytotoxicity on U-937 cells compared with several other cell lines. The inhibitory effects are time- and dose- dependent. The change in cellular morphological features and the results of annexin V/caspase 3 analyses suggest that the induction of apoptosis is responsible for Tan IIA-induced U-937 cell death. Through genome-wide expression profiling, we found that the most significantly regulated gene in response to Tan IIA treatment is chemokine CCL2. These raised two questions: whether or not down-regulation of CCL2 is responsible for cellular apoptosis and how Tan IIA leads to the down-regulation of CCL2.

Chemokines are small heparin-binding proteins that interact with their receptors, regulating many pathophysiological responses in hematological malignancy and inflammatory diseases [21,22]. CCL2 belongs to the CC chemokine family and is produced by a variety of cell types, including monotype, macrophages and many others. As a potent chemoattractant, CCL2 and its receptor, CCR2, play vital roles in regulating the migration and infiltration of monocyte, T lymphocytes and natural killer cells [23]. Recent studies have described the tumor-promoting roles of CCL2/MCP-1 in eosinophilic leukemia EoL-1 cells [22] and breast cancel cells [24-27]. Two molecular mechanisms have also been proposed for CCL2's effects. One is that CCL2 negatively regulates AMP-Activated Protein Kinase to sustain mTOR Complex-1 activation, surviving expression and cell survival in human prostate cancer PC3 cells [27]. The other is that CCL2 activates Gi/Go protein/PLC/PKCδ/ p38 MAPK cascade and PKCδ acts as an anti-apoptotic molecule by regulation of the caspase 3 and caspase 9 [24]. Consequently, it is conceivable that the disruption of CCL2 signaling pathways could promote apoptosis and stunt growth of U-937 cells.

During the course of our study, another report showed that Tan IIA is an agonist of pregnane × receptor (PXR, NR1l2) [20]. We then tested the effect of a PXR antagonist, L-SFN on Tan IIA-treated cells using colony forming unit assay and found that L-SFN significantly mitigate Tan IIA's growth-inhibition effect. Previous studies have also showed that nuclear transcription factor-κ B (NF-κB) regulates the transcription of many inflammatory mediators, including CCL2. Lack of NF-κB activity significantly reduced injury-induced CCL2 expression in astrocytes [28]. Sequence analyses have found NF-κB binding sites on the upstream of CCL2 coding sequences [29]. Furthermore, the interaction of PXR and NF-κB has been described [30]. PXR and NF-κB compete for the RXR to form heterodimer which are required for the transcription of their target genes [31].

**Figure 8:**
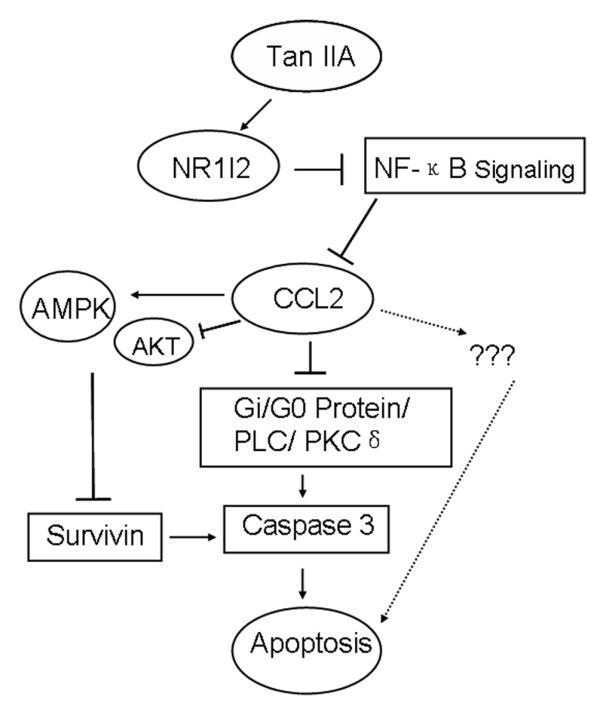
**A proposed model for CCL2-mediated, Tan IIA-induced apoptosis in U-937 cells**.

Integrating above information with our experimental results, we proposed a model explaining Tan IIA-induced, CCL2-mediated apoptosis of U-937 cells (Figure 8). Based on this model, CCL2 is highly expressed in U-937 cells compared with other cells and is critical for the U-937 cell's survival, possibly through the negative regulation of the AMP-Activated Protein Kinase pathway and positive regulation of the Gi/Go protein/PLC/PKCδ/p38 MAPK pathway. The high level expression of CCL2 is maintained by the hyperactivation of NF-κB. Tan IIA is an agonist of PXR, activation of PXR disrupts the high level expression of CCL2 maintained by NF-κB hyperactivation. This leads to the collapse of CCL2's high level expression and consequently promotes the apoptosis of U-937 cells. Additional experiments are needed to further test this model.

## Conclusions

In the current study, we showed that Tan IIA, a diterpene quinone extracted from the root of *Salvia miltiorrhiza*, has significant growth inhibition effect on U-937 cells in vitro through apoptosis induction. Using genome-wide gene expression profiling, we identified hundreds of genes that are significantly up-and down-regulated after Tan IIA treatment. One likely mechanism is that CCL2 is significantly over-expressed in U-937 cells and might play an important role in the survival and proliferation of U-937 cells. Tan IIA activates PXR, which inhibits NF-κB activity, leading to the significantly down-regulation of CCL2 expression by approximately 10-fold, resulting in the apoptosis of U-937 cells.

## Competing interests

The authors declare that they have no competing interests.

## Authors' contributions

CL and MY designed the current study. CL analyzed the GeneChip data. LJW performed the cellular toxicity and apoptosis assays, and carried out the QPCR and ELISA experiments. JQL drafted the manuscript. YQC critically reviewed the manuscript.

## Pre-publication history

The pre-publication history for this paper can be accessed here:

http://www.biomedcentral.com/1472-6882/12/5/prepub

## Supplementary Material

Additional file 1 **Table S1**. List of primers for RT-qPCR. Table S2. List of significantly regulated genes after Tan IIA treatment in U-973 cells. Table S3. List of differentially expressed genes (> 1.5-fold changes) identified from Affymetrix GeneChip experiments that are involved in apoptosis, NF-κB signaling cascade and cell proliferation pathways. Table S4. List of correlations between expression profiles of each gene across the five cell lines and the cell lines' Tan IIA sensitivities to Tan IIA's treatment. Click here for file
